# Urease and Dental Plaque Microbial Profiles in Children

**DOI:** 10.1371/journal.pone.0139315

**Published:** 2015-09-29

**Authors:** Evangelia Morou-Bermudez, Selena Rodriguez, Angel S. Bello, Maria G. Dominguez-Bello

**Affiliations:** 1 School of Dental Medicine, University of Puerto Rico Medical Sciences Campus, San Juan, Puerto Rico, United States of America; 2 School of Medicine, University of Puerto Rico Medical Sciences Campus, San Juan, Puerto Rico, United States of America; 3 New York University School of Medicine, New York, New York, United States of America; 4 University of Puerto Rico Rio Piedras Campus, San Juan, Puerto Rico, United States of America; University of Florida, UNITED STATES

## Abstract

**Objective:**

Urease enzymes produced by oral bacteria generate ammonia, which can have a significant impact on the oral ecology and, consequently, on oral health. To evaluate the relationship of urease with dental plaque microbial profiles in children as it relates to dental caries, and to identify the main contributors to this activity.

**Methods:**

82 supragingival plaque samples were collected from 44 children at baseline and one year later, as part of a longitudinal study on urease and caries in children. DNA was extracted; the V3–V5 region of the 16S rRNA gene was amplified and sequenced using 454 pyrosequencing. Urease activity was measured using a spectrophotometric assay. Data were analyzed with Qiime.

**Results:**

Plaque urease activity was significantly associated with the composition of the microbial communities of the dental plaque (Baseline P = 0.027, One Year P = 0.012). The bacterial taxa whose proportion in dental plaque exhibited significant variation by plaque urease levels in both visits were the family Pasteurellaceae (Baseline P<0.001; One Year P = 0.0148), especially *Haemophilus parainfluenzae*. No association was observed between these bacteria and dental caries. Bacteria in the genus Leptotrichia were negatively associated with urease and positively associated with dental caries (Bonferroni P<0.001).

**Conclusions:**

Alkali production by urease enzymes primarily from species in the family Pasteurellaceae can be an important ecological determinant in children’s dental plaque. Further studies are needed to establish the role of urease-associated bacteria in the acid/base homeostasis of the dental plaque, and in the development and prediction of dental caries in children.

## Introduction

Ureases are large, multisubunit bacterial enzymes that catalyze the hydrolysis of urea into ammonia and carbonic acid, leading to an increase in the local pH [1]. The generation of alkali via the urease pathway is an important virulence factor for some human pathogens, such as *Helicobacter pylori*, and *Ureaplasma ureolyticum*. In the case of *H*. *pylori*, the generation of ammonia via urease contributes to its virulence because it allows the organism to survive in the acidic gastric environment where it can cause gastritis, peptic ulcers and gastric cancer. In the case of *U*. *ureolyticum* the increase in the pH from ureolysis promotes the precipitation of salts in the urine leading to the formation of kidney stones and urinary tract infections [1].

Several oral bacteria, such as *Streptococcus salivarius*, *Actinomyces neaslundii*, and *Haemophilus parainfluenzae*, produce urease enzymes [2–4]. Urea, which is the substrate for these enzymes, is secreted in the oral cavity through the saliva and the gingival crevicular fluid at concentrations that are comparable to those in the plasma, around 3 to 5 mM in healthy individuals [5]. The role of urease in the pathology of oral diseases is somewhat contradictive. High production of ammonia from urease can be toxic to the gingival tissues and can also precipitate the formation of calculus; for these reasons urease may increase the risk for periodontal disease. Conversely, increased alkali production from urease in the dental plaque can help neutralize the acids produced from the glycolytic processing of dietary sugars, and thus prevent the development of dental caries [2, 6, 7]. Recent clinical studies have shown that plaque urease activity is negatively associated with caries experience in adults, [8, 9], and that children with high plaque urease levels may have a lower risk for developing new caries compared to children with low plaque urease [10].

Urease enzymes can play important roles in the physiology of the bacteria that produce them. Urea hydrolysis by ureases generates ammonia, which is a readily assimilated form of nitrogen, and it can thus confer a selective advantage to the ureolytic bacteria during periods of limited nitrogen availability in plaque [11]. The generation of ammonia from urea can also help neutralize the acids produced by the bacteria in dental plaque from the glycolytic processing of sugars, and it can help non-aciduric bacteria survive during periods of excessive acidification [12]. Urease was shown to be important for maintaining the balance of a ten-species biofilm consortium of oral bacteria in a continuous culture biofilm model [9]. A more recent study demonstrated the significant impact of pH on the diversity of microbial communities of caries lesions [13]. However, the exact impact of urease on the ecology of the dental biofilm, which includes more than 700 different bacterial species, has never been demonstrated at the clinical level. Additionally, the organisms that are primarily responsible for the urease activity of the dental biofilm have not been identified.

Based on information summarized previously, the hypothesis of this study was that urease can significantly impact the ecological balance of the dental biofilm, and that this impact can be related to caries status. The objective of this study was to evaluate the impact of urease on the diversity and the composition of the microbial communities of the dental plaque from children as it relates to dental caries, and to identify the organism(s) primarily responsible for this activity. This knowledge could help us find new ways to manipulate these factors in a more effective way in order to establish a healthy ecological balance in the oral biofilms.

## Methods

The study group consisted of 82 samples of supragingival dental plaque that were collected from 44 children as part of a longitudinal study of dental caries [10, 14]. It was a convenience group because it included all available samples from the baseline visit (38 samples) and from the one-year follow-up (44 samples).

Samples were collected during morning hours, between 8 and 10 am. The children were asked to refrain from oral hygiene procedures the night before and the morning of the visit, in order to allow plaque to accumulate. All children were healthy and had not taken antibiotics during the past two months prior to each visit. Dental plaque was collected from all available dental surfaces and pooled into pre-weighed micro centrifuge tubes. The samples remained on ice during collection and were immediately transferred to the adjacent laboratory where they were weighed again to determine the amount of plaque collected. Subsequently, the samples were suspended in 300 μl of 10 mM sodium phosphate buffer, aliquoted, snap-frozen in dry-ice ethanol bath, and stored at -80°C, usually within less than 2 hours from collection time.

Dental exams were performed with an enhanced visual method using a Fiber-Optic Trans-Illumination apparatus (FOTI) (SCHOTT North America Inc., Southbridge, MA). Caries lesions were scored using a combination of visual and FOTI criteria, as described by Cortes, Ellwood and Ekstrand [10, 15]. Dental caries were defined at the d3 level, which included non-cavitated lesions with dentinal involvement. All dental exams were performed by one examiner calibrated as previously described [10]. The study was approved by the Institutional Review Board of the University of Puerto Rico Medical Sciences Campus. A written consent was obtained from the parents or legal guardians of the children. Children participated voluntarily in the study and provided written assent as appropriate for age.

Urease activity in the plaque and saliva samples was measured using a spectrophotometric method, and was expressed as μmoles urea hydrolyzed/min/mg protein as previously described [9, 14].

### Pyrosequencing

DNA was extracted from the samples using the DNeasy Blood and Tissue Kit (Qiagen Co). The region V3–V5 of the *16S rRNA* gene was amplified with the primers 357F/926R (*357F-CCTACGGGAGGCAGCAG*, *926R-CCGTCAATTCMTTTRAGT*) [16] and pyrosequenced in a single multiplexed run on 454 GS FLX+ (Roche Biosciences) at the Sequencing and Genotyping Facility of the University of Puerto Rico. Individual, barcodes were used to identify the sequences obtained from each sample. The primer pair 357F/926R is one of the recommended by the NIH Human Microbiome Project protocols because they amplify all eubacteria. All sequence and related metadata files are available from the Harvard Dataverse Network (doi:10.7910/DVN/RBYNUF).

### Data analysis

The QIIME bioinformatics pipeline [17] was used for the processing and analysis of the metagenomic data. Quality filtering was performed with the following criteria: length between 200 to 1000nt, 0 mismatches, limit of ambiguous bases: 6, homopolymer run limit: 6. Operational Taxonomic Units (OTUs) were defined at 97% similarity. Assignment of taxonomy was performed using the Greengenes reference database [17–19]. A more detailed taxonomic classification of the statistically significant OTUs was subsequently obtained by BLAST against the Human Oral Microbiome Database [20]. Diversity within samples (“alpha diversity”) was measured with Faiths Phylogenetic Distance [21]. Alpha rarefaction plots were used to evaluate alpha diversity as a function of sequencing depth. Diversity between samples (“beta diversity”) was measured with Unifrac distances [22] and evaluated using Principal Coordinate Analysis (PCoA) plots. This analysis was performed using the default even sampling depth suggested by Qiime for each set of samples (3241 for the Baseline samples, and 4533 for the One Year samples). The Permanova test and the Mantel test were conducted on the UniFrac weighed distance matrix in order to determine the effect of urease on the beta diversity of the microbial communities. The ANOVA test with Bonferroni correction for multiple comparisons was used to compare the composition of the plaque samples by urease activity at different taxonomy levels. The g-test (goodness of fit test) with the Bonferroni correction for multiple comparisons was used to compare the frequency of each OTU among different urease and caries groups. Paired t-test was used to compare the abundance of OTUs between the two visits.

## Results

The study group consisted of 41% males and 59% females with average age of 4.9 (±1.19) years at baseline (3 to 6 years). At baseline, 44.7% of the children were free of active caries or restorations (“caries-free”: dmfs = 0) and 55.3% had active caries and/or dental restorations (“caries-experienced”: mean dmfs = 8.72). In the samples from the One Year follow-up visit, 43.2% of the children were caries-free, and 56.8% were caries-experienced (mean dmfs = 12.96).

After passing through quality filters, 214,461 sequences from the baseline samples (average 5,643.7 ± 3,061.7 sequences per sample) and 286,543 sequences from the samples collected one year later (average 6,512.3 ± 4,402.3 sequences per sample) remained for the analysis. The average length of the sequences was 438.9 nt for the baseline samples and 440.7 nt for the one year samples. A total of 2,370 distinct Operational Taxonomic Units (OTUs) were identified in the baseline samples, and 3,178 in the samples from the one-year follow-up visit.

### Diversity within samples (“alpha diversity”)

Alpha rarefaction plots were used to evaluate the diversity within the plaque samples (“alpha diversity”) as a function of sampling depth. No significant differences were observed in the alpha diversity indices (Chao1, Observed Species, Faiths Phylogenetic Distance) by plaque urease, by caries status, or by change in urease levels or caries status (P>0.05). (S1 Fig)

### Diversity between samples (“beta diversity”)

**Fig 1:**
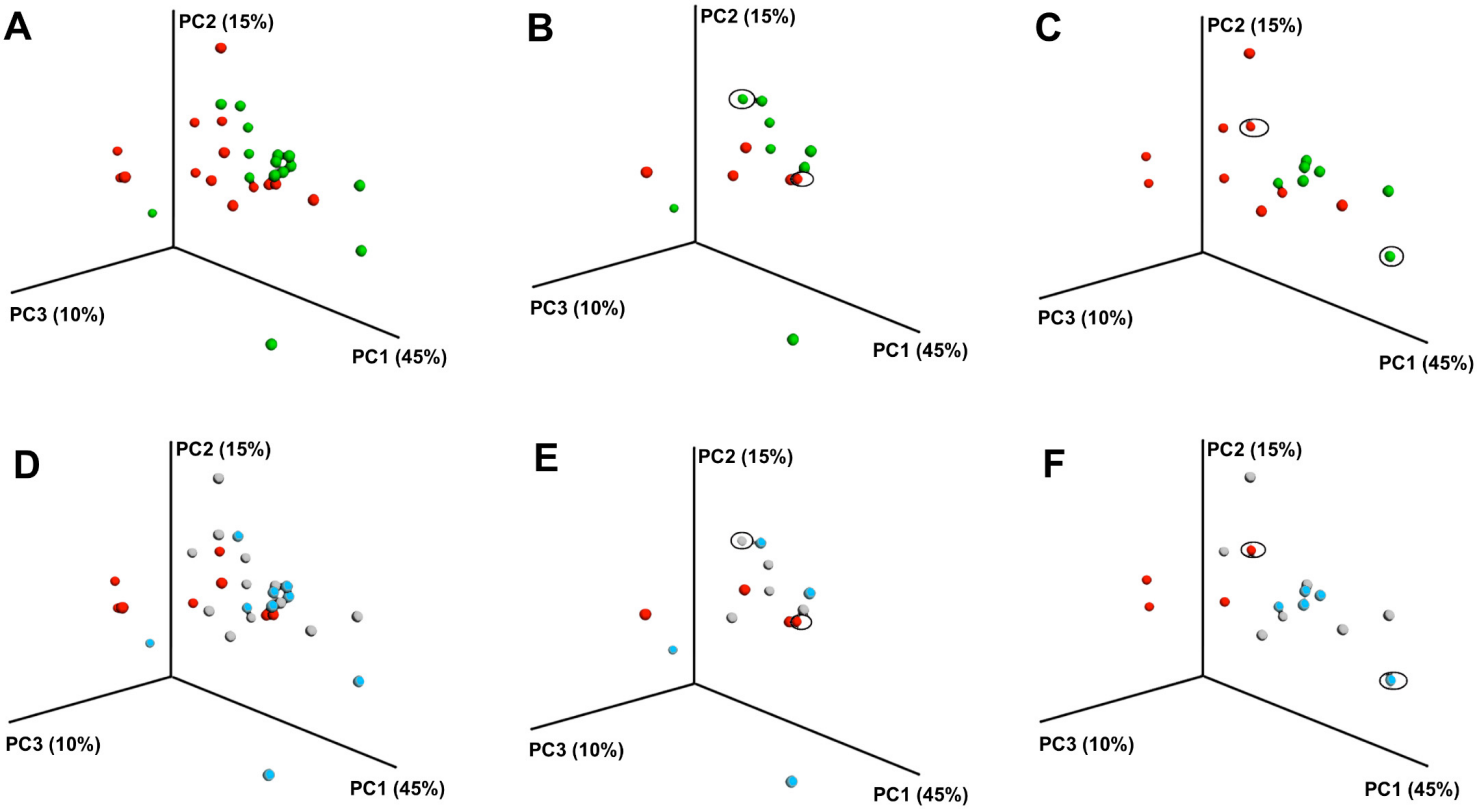
Principal Coordinate Analysis of bacterial communities in Baseline samples of dental plaque from children by plaque urease activity and caries status. Figures A, B and C: samples divided by median urease activity: red- below the median (<2.65 units/mg); green- above the median (>2.65 units/mg). Figures D, E, and F: red- lowest urease quartile (<1.72 units/mg) vs. bleu- highest urease quartile (>4.0 units/mg); light gray: second and third quartile of urease (1.72–4.0 units/mg). A and D: All samples; B and E: Caries-Free (dmfs = 0); C and F: Caries-Experienced (dmfs>0). Circled samples in the Caries-Free groups belong to children who were initially caries free but developed caries within the one-year study period. In the Caries-Experienced group, the circled samples correspond to children who had previous caries but did not develop any new lesions within the one-year study period.

**Fig 2:**
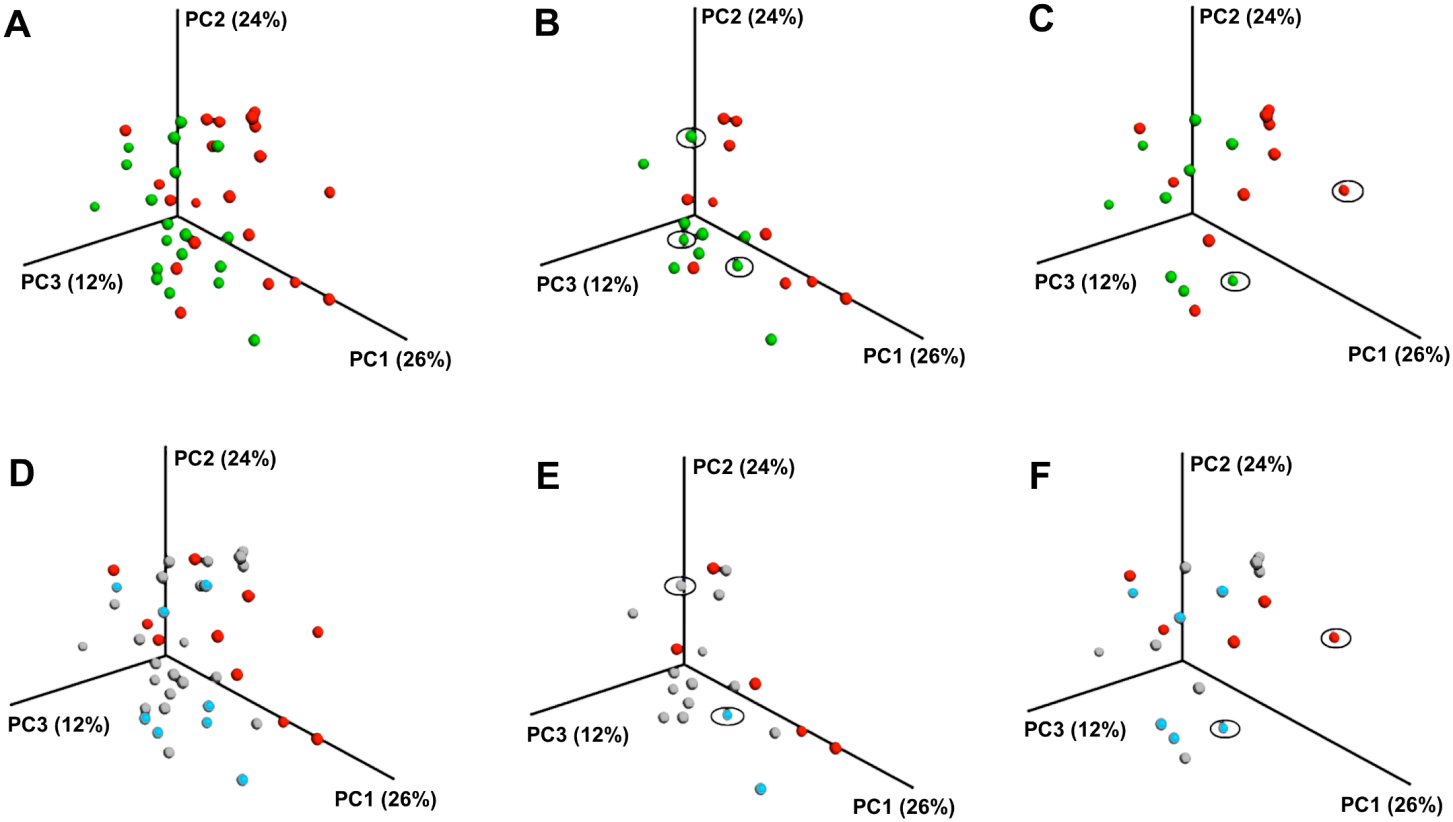
Principal Coordinate Analysis of bacterial communities in One-Year samples of dental plaque from children by plaque urease activity and caries status. Figures A, B and C: samples divided by median urease activity: red- below the median (<2.16 units/mg); green- above the median (>2.16 units/mg). Figures D, E, and F: red- lowest urease quartile (<1.62 units/mg) vs. bleu- highest urease quartile (>2.86 units/mg); light gray: second and third quartile of urease (1.62–2.86 units/mg). A and D: All samples; B and E: Caries-Free (dmfs = 0); C and F: Caries-Experienced (dmfs>0). Circled samples in the Caries-Free groups belong to children who were initially caries free but developed caries within the one-year study period. In the Caries-Experienced group, the circled samples correspond to children who had previous caries but did not develop any new lesions within the one-year study period.

Bacterial diversity between plaque communities (“beta diversity”) was significantly correlated with plaque urease levels (Mantel r = 0.335, P = 0.041 for Baseline samples, and r = 0.298, P = 0.014 for the samples from the One Year follow-up visit). In the PCoA plots samples with plaque urease levels below the median appeared to cluster closer to each other compared to the samples with plaque urease levels above the median (Permanova P = 0.034 for Baseline samples, and P = 0.012 for samples from the One Year visit) (Figs 1A and 2A). When the samples were divided by caries status, the caries-free (Figs 1B and 2B) and the caries-experienced children (Figs 1C and 2C) included both high and low urease samples. Similar findings were observed when the caries status was defined by active caries (lesions in the dentine) instead of overall caries experience (dmfs; data not shown). The clustering pattern by urease was observed in both the caries-free and caries-experienced children but no statistics were performed due to the small number of samples. The findings were also similar when the comparison was focused on the highest and lowest urease quartiles, which had be shown to have the most significant differences in caries risk in our study [10] (Figs 1D, 1E and 1F and 2D, 2E and 2F). No significant differences in beta diversity measures were observed by caries status (P>0.05). (S2 Fig)

### Taxon Summary Analysis

**Fig 3:**
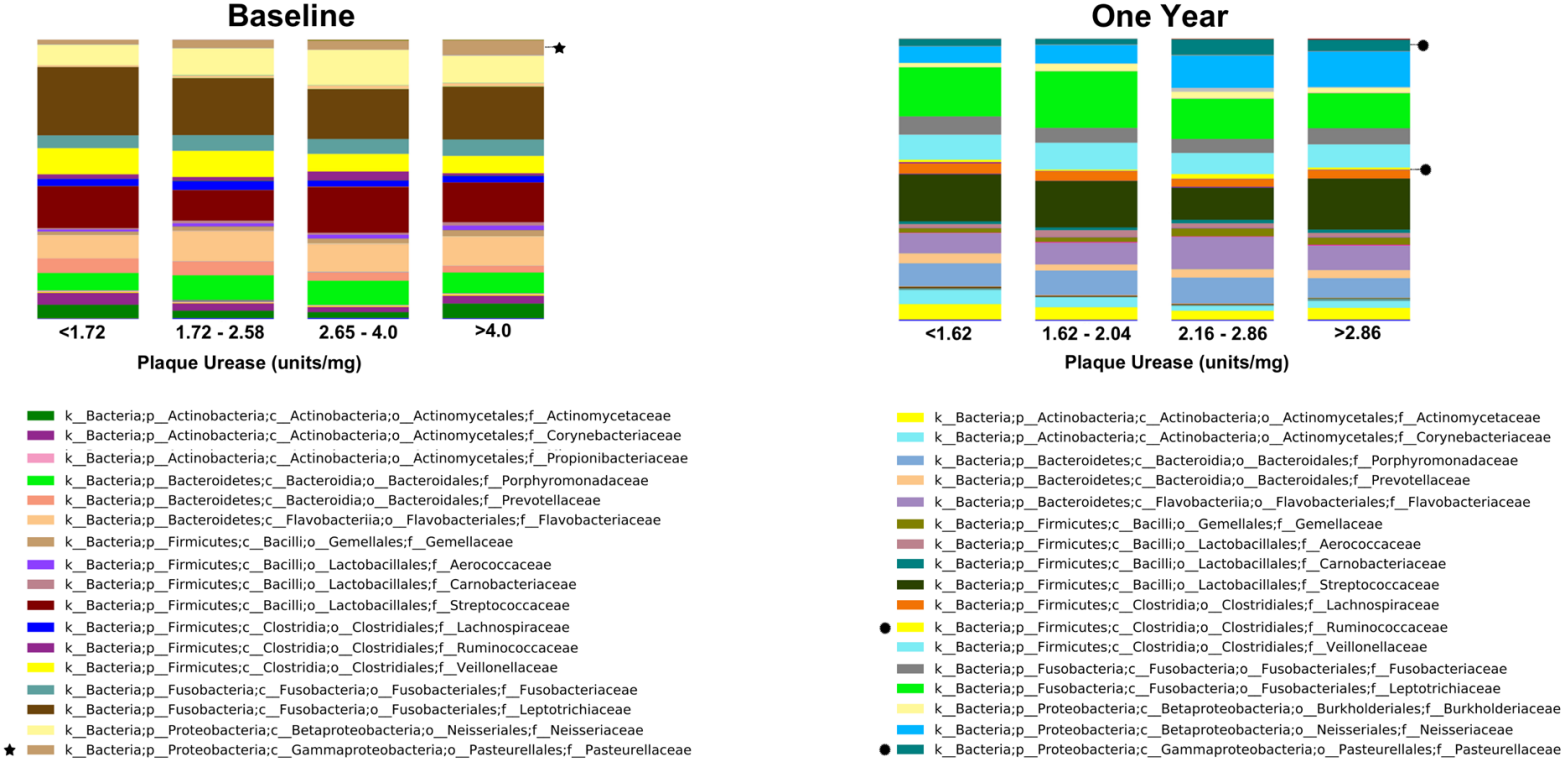
Bacterial taxon summary at Family level for dental plaque samples by plaque urease activity at Baseline and One Year later. *indicates a significant difference in the variance of the bacterial family among the different urease categories after multiple comparisons (ANOVA P<0.05, Bonferroni P<0.05). ·ANOVA P<0.05, Bonferroni P>0.05).

**Fig 4:**
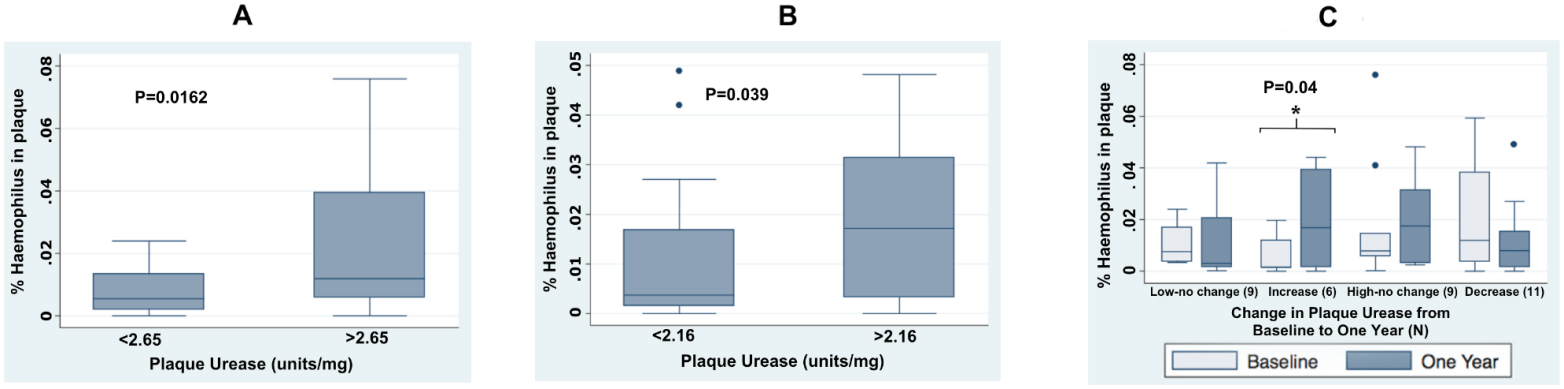
Proportions of bacteria in the genus Haemophilus in the dental plaques from children by plaque urease levels at baseline (A) and one year later (B). Proportions of bacteria in the genus Haemophilus in dental plaques at baseline (light gray) and one year later (dark gray) according to the changes in urease activity between the two visits (C).

Significant variations in the composition of the dental biofilm by plaque urease levels were observed at the taxonomic levels of class and beyond. In the baseline samples, bacterial taxa whose proportion in dental plaque exhibited significant variation by plaque urease levels was the class of Gammaproteobacteria (Bonferroni P = 0.003), order Pasteurellales (Bonferroni P = 0.006), family Pasteurellaceae (Bonferroni P = 0.010) (Fig 3A). At the genus level, the genus Haemophilus, which belongs to the family Pasteurellaceae showed significant variation by plaque urease (ANOVA P = 0.002), but this variation was not significant after correction for multiple comparisons (Bonferroni P>0.05). The same taxonomy groups exhibited significant variation by urease levels (ANOVA P<0.05) in the samples from the One Year follow-up visit up to the Family level, but this variation was not significant after correction for multiple comparisons (Bonferroni P>0.05). The family Ruminococcaceae also showed significant variation by urease levels in these samples (ANOVA P<0.05, Bonferroni P>0.05) (Fig 3B). Bacteria in the genus Haemophilus were significantly more prevalent in samples with high overall plaque urease activity (classified by the median) compared to those with low plaque urease, at both time points (Baseline samples: t-test P = 0.016; One Year samples t-test P = 0.039) (Fig 4A and 4B). Furthermore, the proportion of these bacteria increased significantly in plaque samples in which plaque urease also increased significantly between the two visits (Paired t-test P = 0.04) (Fig 4C).

### OTU-level Analysis

**Fig 5:**
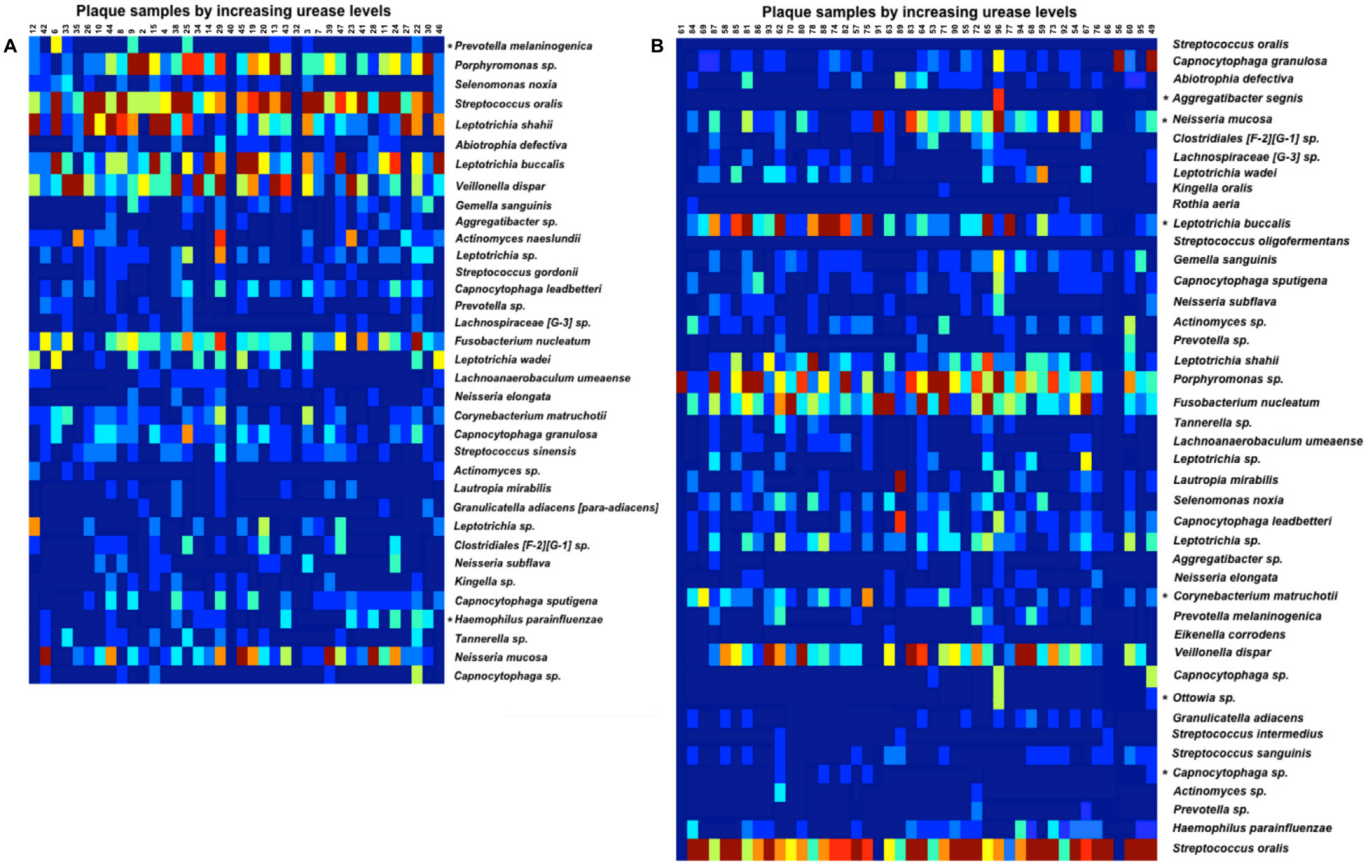
Heatmap and assigned taxonomy of the most representative OTUs (minimum 500 copies) in dental plaque samples at baseline (A) and one year later (B). Samples are arranged by increasing plaque urease levels (left: lowest, to right: highest). * indicates OTUs that exhibit significant difference among samples with urease below the median vs. those with urease levels above the median (Bonferroni P<0.001).

Several OTUs appeared to be significantly more frequent in plaque samples with urease levels above the median, compared to those with urease levels below the median (Bonferroni P<0.001) (Fig 5A and 5B and Table 1). These included *Haemophilus parainfluenzae*, *Neisseria mucosa*, and three low-abundance OTUs classified as *Aggregatibacter segnis*, Ottowia sp., and Capnocytophaga sp. OTUs that became less frequent as plaque urease increased (Bonferroni P<0.001) included *Prevotella Melaninogenica*, *Leptotrichia buccalis*, and *Corynebacterium matruchotii*.

**Table 1:** OTUs whose frequencies in dental plaque of children differ significantly* by: A) plaque urease (above median vs. below median), B) caries experience (dmfs>0 vs. dmfs = 0), and C) active dentine caries (dentine caries present vs. no dentine caries). Taxonomic classification of the OTUs into species was performed by BLAST against the HOMD.

**A) Plaque Urease** **+ change indicates that the OTU is significantly more frequent in the high urease group**			
**Baseline**		**One Year**	
**Species**	Change	Species	Change
*Haemophilus parainfluenzae*	+	*Neisseria mucosa*	+
*Prevotella melaninogenica*	-	*Aggregatibacter segnis*	+
		*Ottowia sp*. *oral taxon 894*	+
		*Leptotrichia buccalis*	-
		*Capnocytophaga sp*. *oral taxon 332*	+
		*Corynebacterium matruchotii*	-
**B) Active Dentine Caries** **+ change indicates that the OTU is significantly more frequent in the caries-active group**			
*Leptotrichia shahii*	+	*Aggregatibacter segnis*	+
*Streptococcus oralis*	+	*Ottowia sp*. *oral taxon 894*	+
*Porphyromonas sp*. *oral taxon 279*	+	*Capnocytophaga sp*. *oral taxon 332*	+
*Capnocytophaga leadbetteri*	+	*Neisseria mucosa*	+
*Leptotrichia sp*. *oral taxon 392*	+	*Capnocytophaga granulosa*	+
		*Leptotrichia shahii*	+
		*Leptotrichia sp*. *oral taxon 219*	+
		*Neisseria subflava*	+
**C) Overall Caries Experience (DMFS)** **+ change indicates that the OTU is significantly more frequent in the caries-experienced group**			
*Leptotrichia shahii*	+	*Aggregatibacter segnis*	+
*Streptococcus oralis*	+	*Ottowia sp*. *oral taxon 894*	+
*Capnocytophaga leadbetteri*	+	*Neisseria mucosa*	+
		*Neisseria subflava*	+
		*Capnocytophaga granulosa*	+
		*Capnocytophaga sp*. *oral taxon 332*	+
		*Abiotrophia defectiva*	-

* g-test with Bonferroni correction for multiple comparisons P<0.001.

OTUs that were significantly more abundant (Bonferroni P<0.001) in plaques from caries-experienced children (dmfs>0), compared to those with no previous caries experience (dmfs = 0), included *Leptotrichia Shahii*, *Streptococcus oralis*, two species of Capnocytophaga (*C*. *leadbetteri* and *C*. *granulosa*), two species of Neisseria (*N*. *mucosa* and *N*. *subflava*), *Aggregatibacter segnis* and *Ottowia sp*. (Table 1). In addition to those, children with active caries had significantly higher numbers of *Porphyromonas sp*., compared to children with no active caries. The only species that was significantly more frequent in caries-active children in both time points was *L*. *Shahii*. The only species that appeared to be significantly less frequent in caries experienced children was *Abiotrophia defectiva*, but only in the one-year samples.

In the samples whose urease activity increased within a year the only OTU that increased significantly corresponded to *H*. *parainfluenzae* (Paired t-test P = 0.041). In the samples whose urease decreased, significant decreases were observed in two low-abundance OTUs classified as genus *Aggregatibacter*, which belongs to the family Pasteurellaceae, one OTU classified as *Capnocytophaga sputnigena* and one in the genus Porphyromonas. This last OTU, however, also decreased significantly in highly ureolytic samples whose activity remained high. OTUs whose numbers changed in an opposite direction to urease activity changes included *Leptotrichia buccalis*, *Prevotella saccharolytica*, *Corynebacterium matruchotii*, and five OTUs that could not be classified into species and belonged to the genera *Clostridiales*, *Tannerella*, *Prevotella*, *Actinomyces*, and *Streptococcus*. None of these differences were statistically significant after correction for multiple comparisons (S1 Table).

Changes in caries status were observed in only four children. Only 2 of the initially caries-free children became caries-active during the one-year period between the two visits, while almost all of the caries-active children except two, developed new lesions. Due to the small number of children who changed caries status between the two visits, no pair-wise comparisons could be made in these groups. In the longitudinal analysis an OTU classified with equal probability as *Streptococcus peroris*, *oralis*, or *mitis* had significant negative correlation with the dmfs index (r: -0.79, P<0.001, Bonferroni P<0.05). Several other OTUs showed significant positive or negative correlations with the various caries indices in the longitudinal analysis (S2 Table), but none of those was significant after correction for multiple comparisons.

## Discussion

The hydrolysis of urea by ureolytic oral bacteria generates alkali, which can have a significant impact on the ecology of the dental plaque and, consequently, on the development of dental caries [6, 7]. In a recent longitudinal study we evaluated the relationship of urease with caries development in children [10], as well as its relationship with other caries risk factors [14]. The findings of this study suggested that high levels of urease in the dental plaque of children could be associated with a reduced risk for developing dental caries. In the present study we used high throughput sequencing to evaluate the hypothesis that urease activity may also affect the diversity and composition of the microbial communities of the dental plaque in children, and that this impact may be related to the children’s caries status. Our findings confirmed the first part of this hypothesis, but not the second; in other words, significant associations were observed between plaque urease levels and microbial community profiles in the dental plaque of the children, but these profiles were not specific to caries-free or caries-active children.

Urease enzymes have been shown to have important physiological functions in the organisms that produce them, such as helping them survive in acidic environments and providing ammonia, which is a readily assimilated form of nitrogen [11, 12, 23, 24]. These important functions can explain, at least in part, the significant association of urease with the diversity measures of the microbial communities of the dental plaque that was observed in this study. In a continuous culture biofilm model, Shu et al. had shown that loss of urease activity from a highly ureolytic constituent resulted in a significant loss of diversity in the biofilm with a predominance of aciduric species like *Streptococcus mutans* and *Lactobacillus rhamnosus*, the lactate-metabolizing *Veillonella dispar*, and the ureolytic *Actinomyces naeslundii*. The remaining five species in this model, *Streptococcus oralis*, *Neisseria subflava*, *Prevotella nigresens*, *Fusobacterium nucleatum*, *and Porphyromonas gingivalis* essentially disappeared from the biofilm in the absence of adequate levels of urease activity [25]. In our study, increased urease activity favored bacteria in the genera of *Haemophilus*, *Aggregatibacter*, *Neisseria*, *Capnocytophaga* and *Ottowia*, while *Leptotrichia*, *Prevotella* and *Corynebacteria* were negatively associated with urease levels. With the exception of Hemophilus, these genera are not known to include ureolytic species; therefore their variability according to urease levels is likely related to changes in pH and nitrogen availability, or it may reflect the indirect result of more complex synergistic or competitive relationships between bacteria in the dental biofilm. Further studies will be needed in order to confirm these observations and to identify the physiologic or molecular mechanisms that directly or indirectly influence the proportions of these organisms in plaque in response to urease levels. The differences between Shu’s in vitro model and our metagenomic data can be due to the fact that natural dental plaque has additional mechanisms besides urease to help bacteria cope with pH fluctuations and with changes in nitrogen availability. Such mechanisms could include the Arginine Deiminase System (ADS), Stickland reactions, and others [7]. However, both studies agree that the impact of urease on the composition of the dental biofilm can be significant.

Previous studies have reported changes in the bacterial diversity of the dental biofilm as caries develops [26, 27]. In this study, we did not observe any significant differences in the diversity measures (alpha diversity or beta diversity measures) of the plaque microbiome by caries status, although certain OTUs appeared to be significantly increased or decreased in caries-experienced or caries-active children. Overall, even though urease activity was shown to be associated with significant ecological changes in the dental plaque, these broader ecological changes were not associated with a specific caries status. An important reason for these findings is that the plaque samples used in this study were pooled from all available teeth, which can mask differences between healthy and carious sites, as well as between sites with different urease activity levels. We do not consider this a limitation of the study, as our objective was to identify broader ecological changes that could possibly be detected before the development of caries and be used a signal of increasing caries activity. The only bacteria that were inversely associated with urease activity but positively associated with caries activity were various species of the genera *Leptotrichia*. These bacteria are becoming of interest as potential cariogenic pathogens, because of their high saccharolytic potential, and their ability to ferment a large variety of mono- and disaccharides to lactic acid [28]. The observation that these bacteria can be positively associated with caries and negatively associated with urease levels encourage the further exploration of their role in the development of dental caries and their potential use as caries risk indicators in children.

Several oral bacteria, such as *Actinomyces naeslundii*, *Streptococcus salivarius*, *Haemophilus parainfluenzae*, *Streptococcus salivarius*, and *Staphylococcus epidermidis* are known to be ureolytic [2–4], but the organisms that are primarily responsible for the high levels of urease activity expressed in the dental biofilm have not been yet identified. In our study, the bacterial taxa that showed a significant and consistent relationship to urease levels in dental plaque were the Gammaproteobacteria, especially the family Pasteurellaceae, genus *Haemophilus* and more specifically *Haemophilus parainfluenzae*. The family of Gammaproteobacteria includes some highly ureolytic human pathogens, such as Klebsiella, *Pseudomonas aeruginosa*, and *Proteus mirabilis* [1]. However, only *H*. *parainfluenzae* can be normally found in the dental plaque. Salako and Kleinberg have previously suggested that this organism is a major contributor to the ureolytic activity of supragingival plaque due to its high numbers and in plaque and its site correlation with high plaque pH and ammonia [2, 3]. Our data support this hypothesis, not only because the genus *Haemophilus* was significantly more abundant in the plaques with high urease activity, but also because its proportions significantly increased in the plaque samples whose urease levels increased between the base line and the one-year follow-up visit.

In summary, this study demonstrated that alkali production from urea could be an important ecological determinant in the dental plaque of children. Species in the family of Pasteurellaceae, in particular *H*. *parainfluenzae*, appear linked to high urease activity responsible for the generation of alkali in the dental plaque, while bacteria in the genus *Leptotrichia* can be negatively associated with urease and positively associated with dental caries. Further longitudinal studies are needed to evaluate in more detail the role of these bacteria in the acid/base homeostasis of the dental plaque, and in the development and prediction of dental caries in children.

## Supporting Information

S1 Fig Rarefaction curves of bacterial diversity within dental plaque samples (alpha diversity) at baseline and one year later by plaque urease and caries status. (TIF)

S2 Fig Principal Coordinate Analysis of bacterial communities dental plaque samples from children by caries status. (TIF)

S1 Table OTUs whose frequencies in dental plaque of children changed significantly (Paired t-test P<0.05) between the two visits according to changes in plaque urease activity. (PDF)

S2 Table OTUs whose frequencies in the dental plaque of children correlated significantly (P<0.05) with different caries outcomes. * Bonferroni P<0.05.
(PDF)
