# Supplementary figures and images for: Urease and Dental Plaque Microbial Profiles in Children
Source: PLoS One. 2015 Sep 29;10(9):e0139315. doi: 10.1371/journal.pone.0139315 (PMC4587978; doi:10.1371/journal.pone.0139315)

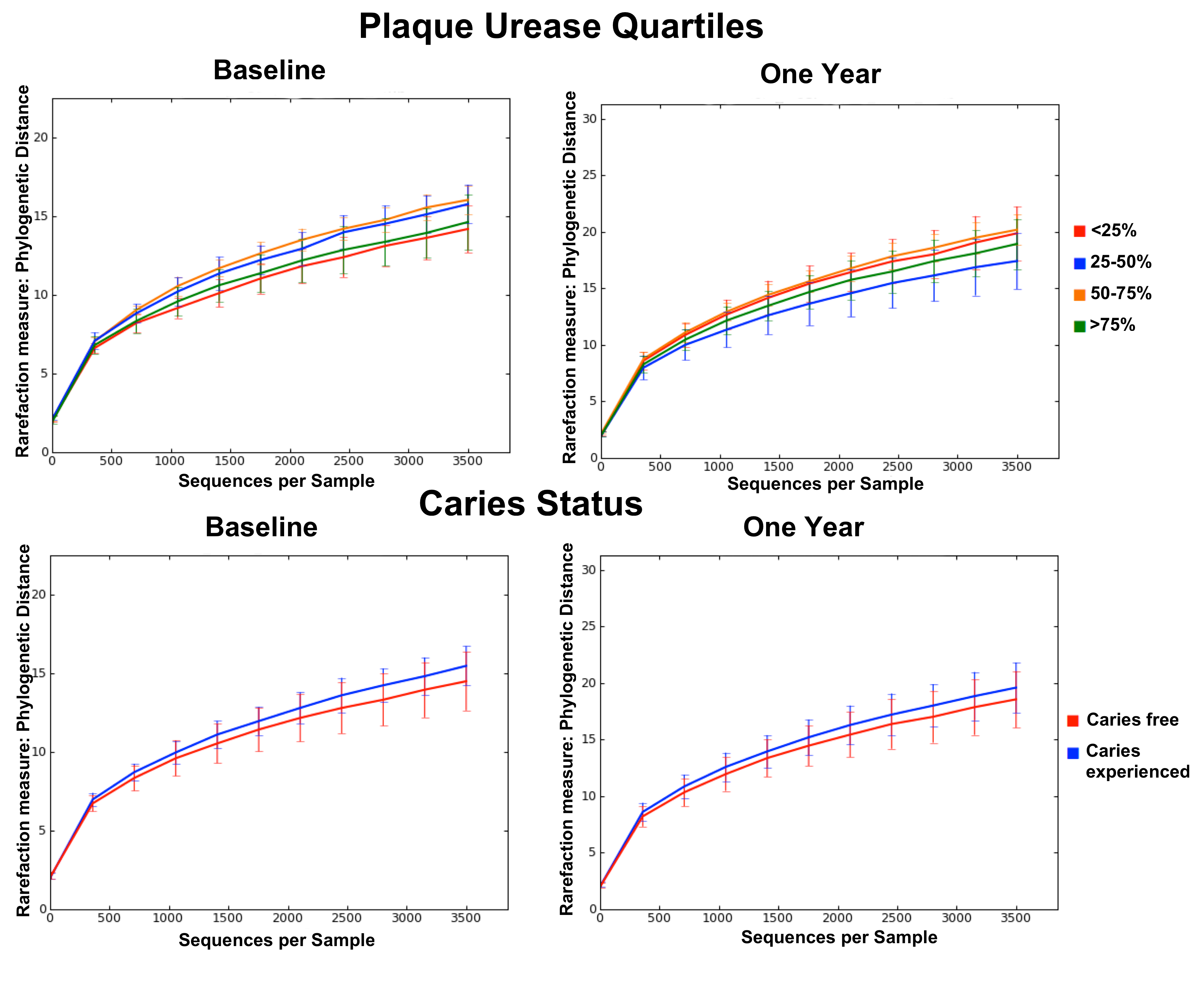

Supplement: S1 Fig — (TIF) [file pone.0139315.s001.tif]

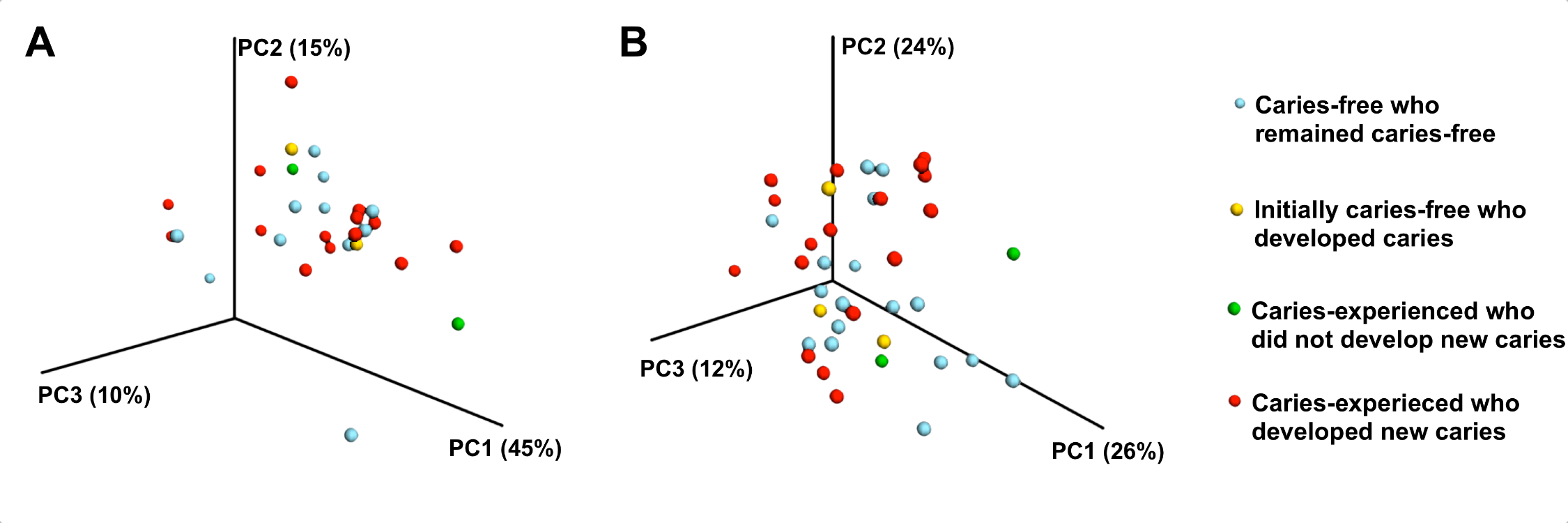

Supplement: S2 Fig — (TIF) [file pone.0139315.s002.tif]
