# Supplementary material for: Urease and Dental Plaque Microbial Profiles in Children
Source: PLoS One. 2015 Sep 29;10(9):e0139315. doi: 10.1371/journal.pone.0139315 (PMC4587978; doi:10.1371/journal.pone.0139315)
Supplement: S1 Table — (PDF) [file pone.0139315.s003.pdf]

S1 Table: OTUs whose frequencies in dental plaque of children changed significantly (Paired t-test P<0.05) between the two visits according to changes in plaque urease activity.

SPECIES	AVERAGE DIFFERENCE	NUMBER PAIRS	PROBABILITY
**Samples in which urease activity increased significantly between visits (Paired t-test P=0.017, N=6 pairs)**			
Bergeyella sp. oral taxon 907	-0.0026	6	0.0031
Streptococcus sinensis	-0.0002	6	0.0259
Clostridiales[F-2][G-1] sp. oral taxon 075	-0.0174	6	0.0293
Leptotrichia buccalis	-0.0728	6	0.0308
Tannerella sp. oral taxon 286	-0.0101	6	0.0399
Haemophilus parainfluenzae	0.0222	6	0.0408
Prevotella sp. oral taxon 317	-0.0176	6	0.0452
Prevotella saccharolytica	-0.0021	6	0.0487
**Samples in which urease activity decreased significantly between visits (Paired t-test P=0.006, N=11 pairs)**			
Corynebacterium matruchotii	0.0002	7	0.0004
Actinomyces sp. oral taxon 170	0.0002	5	0.0056
Aggregatibacter aphrophilus	-0.0004	5	0.0142
Porphyromonas sp. oral taxon 279	-0.0004	7	0.0165
Aggregatibacter sp. oral taxon 898	-0.0039	11	0.0236
Capnocytophaga sputigena	-0.0114	11	0.0242
Streptococcus sp. oral taxon 071	0.0001	7	0.0440
