# Supplementary material for: Urease and Dental Plaque Microbial Profiles in Children
Source: PLoS One. 2015 Sep 29;10(9):e0139315. doi: 10.1371/journal.pone.0139315 (PMC4587978; doi:10.1371/journal.pone.0139315)
Supplement: S2 Table — * Bonferroni P<0.05. (PDF) [file pone.0139315.s004.pdf]

S2 Table: OTUs whose frequencies in the dental plaque of children correlated significantly ($P<0.05$) with different caries outcomes. * Bonferroni $P<0.05$.

Species	r	Probability
**Longitudinal correlation by overall caries experience (dmfs)**		
Streptococcus peroris	-0.7907	<0.0001*
Neisseria flavescens	0.6903	0.0008
Leptotrichia hofstadii	0.7286	0.0014
Actinomyces massiliensis	-0.5011	0.0025
Sneathia amnionii [NV]	-0.7078	0.0046
GN02[G-1] sp. oral taxon 871	0.4876	0.0046
Aggregatibacter aphrophilus	-0.5411	0.0063
Capnocytophaga leadbetteri	-0.6626	0.0071
Fusobacterium nucleatum subsp. Nucleatum	-0.7849	0.0072
Veillonella dispar	0.6082	0.0074
Actinomyces massiliensis	-0.4907	0.0080
Propionibacterium propionicum	-0.3037	0.0090
Actinomyces sp. oral taxon 897	0.5400	0.0095
Leptotrichia sp. oral taxon 879	-0.6608	0.0101
Prevotella sp. oral taxon 472	0.6048	0.0131
Haemophilus haemolyticus	-0.7445	0.0135
Capnocytophaga leadbetteri	-0.2861	0.0141
Veillonella dispar	-0.7337	0.0157
Selenomonas sp. oral taxon 442	-0.2889	0.0161
Capnocytophaga sp. oral taxon 326	-0.2859	0.0164
Leptotrichia sp. oral taxon 225	0.4053	0.0174
Oribacterium sp. oral taxon 372	-0.3340	0.0178
Eikenella corrodens	-0.7198	0.0189
Capnocytophaga leadbetteri	-0.6537	0.0211
Alloprevotella sp. oral taxon 914	-0.3626	0.0215
GN02[G-2] sp. oral taxon 873	0.5676	0.0218
Porphyromonas sp. oral taxon 279	0.2630	0.0246
Veillonella dispar	-0.3788	0.0248
Fusobacterium periodonticum	-0.6907	0.0270

Capnocytophaga sp. oral taxon 903	0.6888	0.0276
Veillonella atypica	-0.2844	0.0276
Campylobacter rectus	0.3299	0.0288
Porphyromonas sp. oral taxon 279	-0.6834	0.0294
Capnocytophaga gingivalis	0.4853	0.0301
Leptotrichia wadei	0.6230	0.0305
Leptotrichia sp. oral taxon 909	0.5963	0.0315
Neisseria flavescens	-0.4579	0.0321
Veillonella parvula	0.3784	0.0327
Veillonella dispar	-0.6720	0.0333
Neisseria elongata	-0.4335	0.0343
Corynebacterium matruchotii	0.6677	0.0349
Neisseria flavescens	0.6592	0.0381
Porphyromonas sp. oral taxon 279	-0.4430	0.0389
Selenomonas sp. oral taxon 442	-0.5991	0.0395
Leptotrichia sp. oral taxon 909	-0.5991	0.0395
Neisseria pharyngis	-0.5380	0.0472
Capnocytophaga granulosa	-0.5370	0.0477
Granulicatella adiacens	-0.2311	0.0492
**Longitudinal correlation enamel caries**		
Actinomyces massiliensis	-0.5482	0.0008
Corynebacterium matruchotii	-0.7296	0.0020
Actinomyces dentalis	-0.3648	0.0033
Kingella oralis	-0.3281	0.0046
Prevotella melaninogenica	-0.3216	0.0055
Aggregatibacter sp. oral taxon 898	-0.5492	0.0066
Fusobacterium periodonticum	0.5607	0.0066
Granulicatella adiacens	-0.3778	0.0096
Streptococcus peroris	-0.7629	0.0103
Porphyromonas sp. oral taxon 279	0.2867	0.0139
Prevotella salivae	-0.6382	0.0141
Prevotella sp. oral taxon 472	-0.6076	0.0163
Gemella morbillorum	-0.4908	0.0174

Kingella oralis	-0.3256	0.0185
Veillonella dispar	-0.3933	0.0194
Eikenella corrodens	0.6174	0.0246
Leptotrichia sp. oral taxon 219	-0.5880	0.0270
Leptotrichia hongkongensis	-0.6301	0.0281
Veillonella sp. oral taxon 780	-0.5367	0.0321
Actinomyces massiliensis	-0.4047	0.0327
Actinomyces sp. oral taxon 180	-0.2499	0.0330
Selenomonas sp. oral taxon 442	-0.2541	0.0351
Streptococcus peroris	-0.5226	0.0378
Veillonella dispar	-0.5922	0.0425
Streptococcus sanguinis	0.5109	0.0432
Selenomonas sp. oral taxon 442	-0.5901	0.0434
Cardiobacterium valvarum	0.3328	0.0442
Capnocytophaga gingivalis	0.5387	0.0469
Prevotella maculosa	-0.2594	0.0472
**Longitudinal correlation by dentine caries**		
Veillonella dispar	-0.8529	0.0017
Porphyromonas sp. oral taxon 279	0.3472	0.0026
Leptotrichia sp. oral taxon 217	0.7430	0.0056
Neisseria elongata	0.6553	0.0059
Veillonella dispar	0.6188	0.0062
Capnocytophaga sp. oral taxon 336	0.4459	0.0093
Porphyromonas sp. oral taxon 284	-0.4007	0.0104
Veillonella dispar	-0.4269	0.0105
Porphyromonas sp. oral taxon 279	-0.5327	0.0107
Corynebacterium matruchotii	0.7478	0.0129
Capnocytophaga leadbetteri	-0.6125	0.0152
Neisseria oralis	-0.6718	0.0167
Actinomyces massiliensis	-0.4067	0.0170
Actinomyces massiliensis	-0.4451	0.0176
Capnocytophaga sp. oral taxon 326	-0.2815	0.0182
Neisseria elongata	0.5362	0.0218

Capnocytophaga leadbetteri	-0.2646	0.0237
Eikenella sp. oral taxon 011	-0.5250	0.0253
Veillonella parvula	0.3911	0.0269
Capnocytophaga leadbetteri	-0.6238	0.0302
Streptococcus peroris	-0.5279	0.0356
Veillonella atypica	-0.2686	0.0380
Kingella oralis	-0.6558	0.0395
Leptotrichia sp. oral taxon 909	0.5727	0.0408
Fusobacterium sp. oral taxon 203	0.5157	0.0409
Capnocytophaga leadbetteri	-0.5507	0.0413
Lautropia mirabilis	0.3738	0.0418
Capnocytophaga gingivalis	0.4565	0.0431
Veillonella dispar	-0.6433	0.0448
Neisseria flavescens	0.4505	0.0462
Tannerella sp. oral taxon 286	0.3666	0.0463
GN02[G-1] sp. oral taxon 871	0.3525	0.0478
Fusobacterium sp. oral taxon 370	0.2586	0.0480
Leptotrichia hongkogenesis	0.3180	0.0485
